# Polyp in concha bullosa: a case report and review of the literature

**DOI:** 10.1186/1746-160X-2-11

**Published:** 2006-05-08

**Authors:** Alper Nabi Erkan, Tuba Canbolat, Cem Ozer, Ismail Yilmaz, Levent N Ozluoglu

**Affiliations:** 1 Baskent University Faculty of Medicine, Department of Otorhinolaryngology, 2. Cadde No:72/2, Bahcelievler 06490, Ankara, Turkey; 2 Baskent University Faculty of Medicine, Department of Pathology, 12. Sokak No:4/1, Bahcelievler 06490, Ankara, Turkey

## Abstract

Polyp originating within a concha bullosa is uncommon; we report only the third such case in the English literature. A 45-year-old man presented with nasal obstruction and headache. Examination of the nose revealed right septal deviation and a hypertrophic left middle concha. Computed tomography confirmed right septal deviation and identified left concha bullosa with thickening of the mucosa covering this lesion. The lateral lamella of the affected turbinate was removed and a mass was excised. Histopathologic examination of the excised mass revealed polypoid hyperplasia. The rare finding of polyp in concha bullosa is discussed with a review of the literature. In any case of concha bullosa, computed tomography images should be carefully evaluated before surgery to check for other pathologies that might have arisen within the lesion.

## Background

Concha bullosa is a cystic distension of the middle nasal concha. This is a common anatomic variation of the middle turbinate, however, polyp formation within concha bullosa is rare. The first documented case of polyp in concha bullosa was noted by Yanagisawa [1] in his book "*The Atlas of Rhinoscopy: Endoscopic Sinonasal Anatomy and Pathology*". Mirante *et al*. [2] reported the second case in a 38-year-old man. Here we describe a concha bullosa polyp in a 45-year-old man. The clinical presentation, radiological and endoscopic findings, and management approach for this case are discussed.

## Case presentation

**Figure 1:**
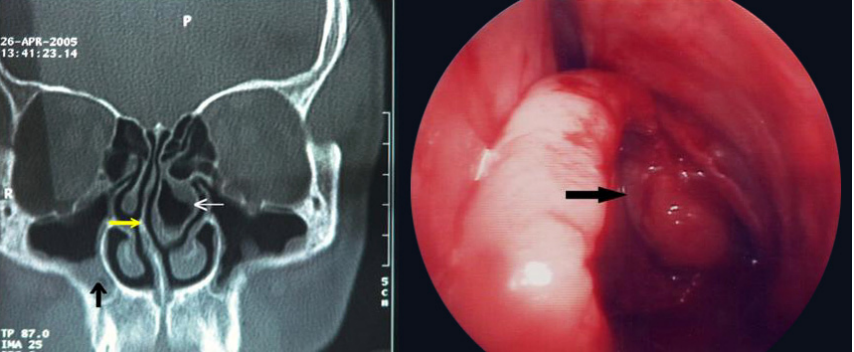
Left: Coronal computed tomography image shows right septal deviation (yellow arrow), left concha bullosa (thick white arrow), thickening of the mucosa covering the concha bullosa, and bilateral maxillary sinusitis (vertical black arrow). Right: Intraoperative views of the polyp in the left concha bullosa. (black arrow shows polyp)

**Figure 2:**
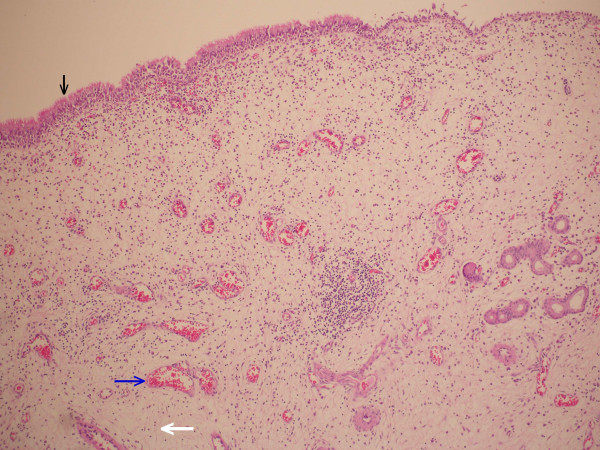
Histopathologic examination of the excised specimen revealed an inflamed polypoid mass covered with respiratory epithelium (vertical black arrow) and surrounded by edematous stroma (bold white arrow) and dilated vessel (blue arrow) (Haematoxylin and Eosin stain, magnification × 100).

A 45-year-old man presented to our clinic with complaints of nasal obstruction and headache. He had had these problems for 4 years. There was no history of nasal trauma or nasal allergy. Nasal examination revealed right septal deviation and a hypertrophic left middle concha. The patient was otherwise healthy and results of routine laboratory tests were normal. Computed tomography (CT) showed right septal deviation, left concha bullosa, and thickening of the mucosa on the inner aspect of the concha bullosa (Figure 1 left). Septoplasty was performed under general anesthesia and the lateral and medial lamellae of the concha bullosa were separated. The lateral lamella was excised and a polypoid mass originating from the inner mucosal surface of the concha bullosa was removed (Figure 1 right). Histopathologic examination of the mass revealed polypoid hyperplasia (Figure 2). The postoperative course was uneventful. Six months after the operation, the patient was free of nasal complaints.

## Discussion

The middle turbinate lies medial to a number of important sinus structures, including the anterior ethmoid air cells, the maxillary sinus ostium, the nasofrontal duct, and the uncinate process. The average length of the middle concha in humans is 40 mm, and the average anterior and posterior thicknesses of this structure are 14.5 mm and 7 mm, respectively [3]. The turbinates help to warm, humidify and filter inspired air [4]. All are covered in a mucosal layer composed of pseudostratified ciliated columnar respiratory epithelium.

Concha bullosa occurs when the middle turbinate becomes pneumatized. As noted, this condition is a very common anatomic variation. This pneumatization results when ethmoid air cells migrate to the middle concha. Zinreich *et al*. [5] used coronal CT to evaluate 320 patients for sinus disease, and found that 34% exhibited concha bullosa on at least one side.

Cases of concha bullosa are categorized as one of three types based on the specific site of pneumatization: 1) lamellar type (vertical lamella pneumatized); 2) bulbous type (inferior portion of turbinate pneumatized; 3) extensive (large) type (vertical lamella plus inferior turbinate pneumatized) [6]. Bolger *et al*. [6] studied anatomic variations of the paranasal sinuses in 202 patients based on CT images, and observed lamellar-type concha bullosa in 46.2% of the cases, bulbous-type concha bullosa in 31.2%, and extensive concha bullosa in 15.7%. The degree of pneumatization determines the severity of symptoms. The lamellar type usually does not cause severe symptoms, whereas the bulbous and extensive forms typically are symptomatic [7]. The most common symptoms are nasal obstruction and facial pain. If the concha bullosa obstructs the middle meatus, the patient may develop sinusitis. Aktas *et al*. [8] found a statistically significant relationship between unilateral concha bullosa and nasal septal deviation, but detected no associations between unilateral or bilateral concha bullosa and sinusitis, or between bilateral concha bullosa and nasal septum deviation.

Most polyps in the nasal cavity develop from the mucosa of the anterior ethmoidal sinus, the contact areas of the uncinate process, and the middle turbinate [9]. These structures are exposed to more air turbulence than other nasal structures. As a result, irritants are more likely to be deposited and trigger inflammation in the mucosa of these regions, promoting polyp development [10]. Polyp formation in a concha bullosa is uncommon. In both of the 2 previously reported cases, the polyp arose from the inner surface of the mucosa covering the concha bullosa. We do not know what caused the polypoid hyperplasia in our case, but inflammation is suspected to promote such lesions.

Polyp in concha bullosa can be diagnosed with CT. The images show mucosal thickening and polypoid tissue within the concha bullosa. The symptoms associated with such polyps cannot be distinguished from those that characterize concha bullosa alone. The treatment for polyp formation in all types of concha bullosa is excision of the lateral lamella of the concha bullosa followed by polyp excision.

In summary, this article describes a rare case of polyp originating from a concha bullosa. Only two similar cases have been reported previously. In any case of concha bullosa, CT images should be carefully evaluated before surgery to check for other possible pathologies within the lesion.

## Competing interests

The author(s) declare that they have no competing interests.

## Authors' contributions

A.N.E has drafted and prepared the manuscript. T.C carried out the histological evaluation. CO carried out the review of the patient's medical record in order to collect all the available information. I.Y and L.N.O were involved in revising the article for intellectual content details. All authors read and approved the final manuscript.
